# Smoking cessation to prevent death and tuberculosis recurrence after treatment: A prospective cohort study with a seven-year follow-up in China

**DOI:** 10.7189/jogh.14.04187

**Published:** 2024-09-06

**Authors:** Haoxiang Lin, Lixin Xiao, Yongming Chen, Xianglin Zeng, Xiaoxu Zhang, Yan Lin

**Affiliations:** 1 Institute for Global Health and Development, Peking University, Beijing, China; 2 Clinical Department, Xingguo County Tuberculosis Dispensary, Xingguo, China; 3 Clinical Department, Ningdu County Tuberculosis Dispensary, Ningdu, China; 4 Beijing Fengtai You’anmen Hospital, Beijing, China; 5 International Union against Tuberculosis and Lung Diseases, Beijing, China

## Abstract

**Background:**

Although there is consistent evidence that smoking is a risk factor associated with tuberculosis (TB), whether smoking cessation improves treatment outcomes and reduces the risk of TB recurrence remains understudied.

**Methods:**

We conducted a prospective cohort study with a seven-year follow-up in China. We recruited newly-diagnosed TB patients and classified them as non-smokers, ex-smokers, and current smokers. Current smokers were invited to participate in a smoking cessation intervention programme. We used a Cox proportional hazards model to assess the risk of death among TB patients and the risk of recurrence among successfully treated patients.

**Results:**

In total, 634 (79.2%) patients completed anti-TB treatments and 115 (14.4%) patients died. We confirmed the existence of a dose-response relationship between smoking frequency and the risk of TB recurrence (the slope of the fitted line >0; *P* < 0.05). Compared to those who continued smoking, the risk of death and recurrent TB for the patients who quit smoking during treatment decreased. The HR of mortality for smokers who smoked 30 or more cigarettes was 2.943 (95% confidence interval (CI) = 1.035–8.368), while the HR of mortality for those who smoked 30 or more cigarettes, but quit during treatment was 2.117 (95% CI = 1.157–3.871). However, the risk of recurrence remained high for ex-smokers who had a smoking history of 25 years or more.

**Conclusions:**

Our study provides further evidence supporting the World Health Organization’s call for co-management of smoking and other risk factors as part of routine TB treatment.

Tuberculosis (TB) is the second leading cause of death from infectious diseases, with most deaths occurring in low- and middle-income countries. Despite a globally decreasing trend up to 2019, the total number of TB deaths increased in 2020 and 2021 [1]. This highlights a need for urgent action to mitigate the current trend of TB deaths in order to achieve the 2030 global TB target set by the United Nations and the World Health Organization (WHO) [1].

Smoking is a known major driver of TB epidemics and is associated with a high risk of poor treatment outcomes and recurrence [2-4]. TB patients have been found to have a greater prevalence of smoking compared to the general population [5]. Smoking also increases the risk of TB infection by 2–2.5 times and is associated with increased TB mortality [6,7], with estimates suggesting that it accounts for 20% of the global TB incidence and 17% of TB treatment failures [8,9]. This is not only due to its direct effects on lung health, but also because it is often associated with other harmful behaviours, such as excessive alcohol consumption [10]. This strong link between smoking and TB has led to the recognition of the need to reduce smoking among TB patients – especially as they are also more likely to quit smoking during TB treatment, particularly in the early stages, presenting an ideal opportunity to support smoking cessation [5].

Although there is consistent evidence that smoking is a risk factor associated with TB, previous studies have primarily focussed only on their epidemiological and biological links to one another. In contrast, whether smoking cessation improves treatment outcomes and reduces the risk of TB recurrence remains understudied. One Cochrane review analysing the impact of smoking cessation on TB treatment outcomes concluded that there is a lack of high-quality evidence on the effectiveness of cessation intervention in improving TB treatment outcomes [7]. Another systematic review published by the International Union Against Tuberculosis and Lung Disease reached the same conclusion and called for additional studies to test how smoking cessation interventions impact patients beyond smoking cessation effects [10].

Until recently, few studies evaluated the effect of smoking cessation on health outcomes during treatment for TB; most found significant positive correlations between smoking cessation and better TB outcomes, including a higher treatment completion rate, lower TB relapse rate, and better quality of life [11,12]. For instance, a short-term follow-up study found that compared with non-quitters, those who quit had better TB outcomes and treatment completion rates (91% vs 80%; *P* < 0.001), as well as a higher sputum conversion rate at week 9 (91% vs 87%; *P* = 0.036) and lower mean TB clinical scores at six months [11]. Additionally, one long-term study found that patients who successfully quit smoking during TB treatment had a lower risk of recurrence than those who continued smoking [12]. However, the findings from this study were limited by an imperfect categorisation of smokers in the analysis – a limitation which could be mitigated by more clearly distinguishing between the effects observed in different subgroups of smokers.

Based on available literature, we hypothesised that, compared to those who continued smoking, the risk of death and recurrent TB for the patients who quit smoking during treatment might decrease. Therefore, we conducted a prospective cohort study with a seven-year follow-up in China to assess whether smoking status and cessation support during anti-TB treatment have long-term effects on reducing the risk of death among newly diagnosed TB patients and the risk of recurrence among successfully treated patients. As there are very few published studies on this topic, we believe that our findings could provide robust long-term follow-up results, thereby offering comprehensive outcomes beyond smoking cessation and enabling detailed comparisons between current smoking behaviour and smoking history through stratified analysis.

## METHODS

### Study design and participants

We conducted a prospective cohort study in two public health TB clinics in Xingguo County and Ningdu County, two agricultural counties in the Jiangxi Province in southeast China, with a total population of 1.67 million. There was only one public health TB clinic situated in the county town. Patients who visited the TB clinics were either self-referred due to experiencing symptoms indicative of TB or were referred by other health care institutions as potential TB suspects. Eligible participants were TB patients who were consecutively registered at the two TB clinics between 1 March and 31 August 2010.

### Procedure

We set our TB diagnosis criteria according to the WHO and China National TB Program (NTP) guidelines [13,14]. Treatment regimens and anti-TB drug formulations were administered according to WHO recommendations and NTP guidelines for presumed drug-sensitive TB. The standardised treatment regimen consisted of two months of isoniazid, rifampicin, pyrazinamide, and ethambutol administered thrice per week, followed by four months of thrice-weekly isoniazid and rifampicin. Patients enrolled in the routine TB programme were eligible for a six-month drug-sensitive treatment regimen, except for certain extra-pulmonary TB patients who might require an extended treatment period beyond the initial six months. According to the local practice, directly observed therapy was implemented throughout the anti-TB treatment by village doctors, other medical staff, family members, or others. Patients were instructed to visit the same TB clinics each month for necessary examinations and to collect their TB medications for the following month's treatment.

After recruitment, participants had to complete a baseline questionnaire that collected information for the initial assessment. This included demographic details, TB diagnosis, treatment regimen, directly observed therapy, smoking status, home address, and telephone number. A list of patients stratified by their home township was distributed to the health workers at the public health TB clinics and township disease control staff for review during the monthly disease control meetings. These patients were monitored for recurrence each year using the annual TB notification register and were visited in their communities by township disease control staff or village doctors.

The International Union Against TB and Lung Diseases conducted a training session on the importance of cessation support and how to provide brief advice to smokers. The Union's guideline ‘Tobacco Cessation Interventions for TB Patients: A Guide for Low-Income Countries’ was translated into the local language and provided to the trained staff. Immediately after their TB diagnosis, current smokers were given brief information about the harmful effects of tobacco and asked if they were willing to quit smoking. Those willing to quit were invited to participate in a smoking cessation intervention programme offered by their TB service providers. At the time of TB registration, patients were provided with information on the harmful effects of smoking and given brief cessation advice. This advice was reinforced during each visit to the TB dispensary. Since TB patients collected their medication monthly from the same clinics, current smokers received cessation advice through face-to-face interactions every month. Further details on the cessation intervention have been published elsewhere [5].

### Measures

We defined TB recurrence as the presence of a new episode of TB in a TB patient who was declared cured or had completed treatment and remained TB-free for a minimum of six months after the end of the most recent anti-TB treatment. Patients were not restricted by smoking status during recruitment at baseline. Initially, we categorised all TB patients as non-smokers, ex-smokers, or current smokers. By the end of the six-month TB treatment, we further classified current smokers into two categories based on their smoking cessation outcomes: those who quit smoking during TB treatment, defined as patients who were current smokers at the time of TB registration but successfully quit smoking during anti-TB treatment, and those who continued smoking during TB treatment, defined as patients who were current smokers at the time of TB registration but continued smoking during anti-TB treatment, regardless of their participation in the cessation intervention.

### Statistical analysis

We used descriptive statistics to summarise our participants’ characteristics. We otherwise used a Cox proportional hazards model for the main analysis, whereby the occurrence of either death in TB patients or recurrence in those successfully treated during the seven-year follow-up period was the dependent variable, and their smoking status was the independent variable. We calculated adjusted hazard ratio (aHR) for the risk of disease incidence and 95% confidence intervals (CIs) via multivariate analyses. We included age, type of TB, smear test, pleurisy and patient management as control variables. The mean number of cigarettes smoked in each category, along with the hazard ratios (HR), were plotted with straight lines of best fit. The slopes of these lines were expressed in terms of changes in the mean number of cigarettes smoked and the corresponding (HR).

We considered a *P*-value <0.05 as statistically significant. All the statistical analyses were conducted using SPSS, version 19.0 (IBM Corp., Armonk, New York, USA). No imputation was performed. The Cox proportional hazards assumption was confirmed by survival curves, while the normality test was confirmed by Kolmogorov-Smirnov analysis. To evaluate how smoking amount affected the results, current smokers were further stratified by daily smoking 15 or fewer cigarettes, 16–29 cigarettes, and 30 or more cigarettes.

We based our sample size calculation was based on Cox regression and checked it using the PASS software (NCSS, LLC, Kaysville, Utah, USA). We hypothesised that the recurrence rate is about 10%; we used a value of 0.80 (β = 0.20) for power and 0.05 for alpha. Considering 10% of deaths and 9% lost to follow-up and other administrative reasons, we calculated that we would need to enroll at least 450 patients.

### Ethical considerations

This research was approved by health authorities at each of the implementation sites. The Ethics Advisory Group of the International Union Against Tuberculosis and Lung Disease, Paris, France, formally approved this study (EAG number: 7/17). Besides the ethical approval and patient consent agreement, the patients were provided with information on the benefits of quitting smoking and were informed that participation in the cessation programme was entirely voluntary, and they could withdraw at any time.

### Quality assurance

Medical staff performed the baseline assessment, while two experts simultaneously performed data entry. TB recurrences were confirmed by medical institutions and documented in the local disease surveillance system.

## RESULTS

We recruited 800 newly diagnosed TB patients, including 572 men and 228 women, all of whom had received WHO-recommended anti-TB treatment for six months. Overall, 115 (14.4%) patients died in the seven-year period of our study. Of the 634 patients (79.2%) who completed their anti-TB treatments, 96 (15.1%) had TB recurrence, while 21 (3.31%) were lost to follow-up.

Most participants were men (71.5%) and under 65 years of age (**Table 1**). The majority had smear-negative TB (63.4%), and 93.1% of them had pulmonary TB. Additionally, 30.6% of the participants were current smokers at the time of TB registration. Among them, 23.8% successfully quit smoking during the siox-month TB treatment period.

**Table 1:** Baseline demographics and key variables (n = 800)

Variables by category	n (%)
Sex	
*Women*	228 (28.5)
*Men*	572 (71.5)
Age in years	
*≤44*	375 (46.9)
*45–64*	286 (35.8)
*≥65*	139 (17.4)
Smear test	
*Positive*	271 (33.9)
*Negative*	507 (63.4)
Extra-pulmonary TB	22 (2.8)
Smoking status	
*Non-smoker*	433 (54.1)
*Ex-smoker*	123 (15.4)
*Quit smoking during TB treatment*	190 (23.8)
*Continued smoking during TB treatment*	54 (6.8)
Type of TB	
*Pulmonary TB*	745 (93.1)
*Others*	55 (6.9)
Have pleurisy	
*Yes*	77 (9.6)
*No*	723 (90.4)

Cs –continued smoking during TB treatment, QS – quit smoking during TB treatment, TB – tuberculosis

**Figure 1:**
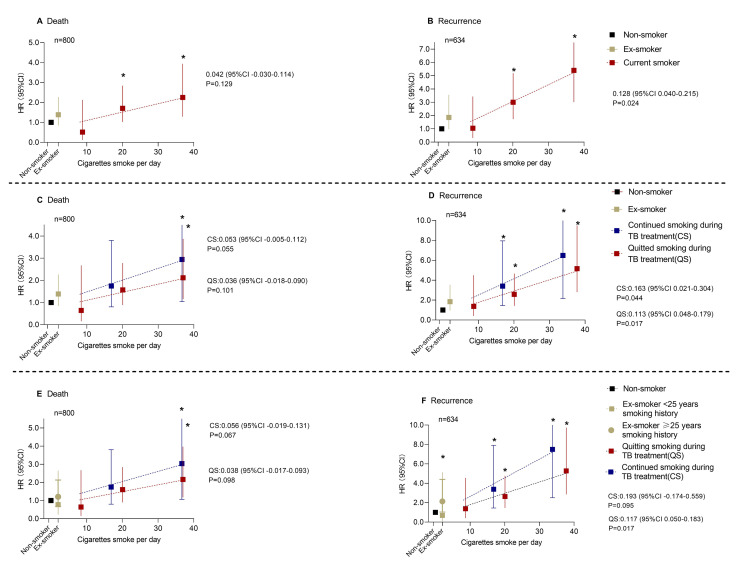
Association between smoking status, death, and TB recurrence. **Panel A.** Association between smoking status and death, stratified by non-smokers, ex-smokers, and smoking status of current smokers. **Panel B.** Association between smoking status and recurrence, stratified by non-smokers, ex-smokers, and smoking status of current smokers. **Panel C.** Association between smoking status and death, stratified by non-smokers, ex-smokers, and smoking status of current smokers. **Panel D.** Association between smoking status and recurrence, stratified by non-smokers, ex-smokers, and smoking status of current smokers. **Panel E.** Association between smoking status and death, stratified by non-smokers, ex-smokers, and smoking status of current smokers. **Panel F.** Association between smoking status and recurrence, stratified by non-smokers, ex-smokers, and smoking status of current smokers.

We performed a multivariate analysis of the association between baseline smoking status and both mortality and recurrence after treatment completion. Th eCox proportional hazards analysis showed a significant increase in the risk of TB mortality and recurrence with higher smoking frequency (**Figure 1**, panels A and B, **Table 2**). This increase was especially pronounced in individuals who smoked 16 or more cigarettes per day. For instance, compared to non-smokers, the aHR for mortality was 1.707 (95% CI = 1.026–2.841, *P* = 0.040) in those who smoked 16-29 cigarettes per day and 2.253 (95% CI = 1.290–3.935, *P* = 0.004) in those who smoked 30 or more cigarettes per day.

**Table 2:** aHRs for baseline smoking and risk of death and TB recurrence*

	Death		Recurrence	
**Variables by category**	**aHR (95% CI)**	***P*-value**	**aHR (95% CI)**	***P*-value**
Smoking status				
*Non-smoker*	ref		ref	
*Ex-smoker*	1.387 (0.849–2.267)	0.192	1.858 (0.969–3.561)	0.062
*Smokes ≤15 per day*	0.513 (0.124–2.129)	0.358	1.044 (0.317–3.433)	0.944
*Smokes 16–29 per day*	1.707 (1.026–2.841)*	<0.05	3.000 (1.734–5.189)	<0.05
*Smokes ≥30 per day*	2.253 (1.290–3.935)*	<0.05	5.402 (3.017–9.672)	<0.05
Omnibus tests	χ^2^ = 112.084; *P* < 0.001		χ^2^ = 81.664; *P* < 0.001	

aHR – adjusted hazard ratio, CI – confidence interval, ref – reference

*We included age, type of TB, smear test, pleurisy, and patient management as control variables.

To examine whether smoking cessation during anti-TB treatment had a positive effect on health by improving survival and reducing the risk of recurrence, we stratified current smokers at baseline by whether they quit smoking during treatment and conducted the same regression again. Compared to those who continued smoking, the risk of death and recurrent TB for the patients who quit smoking during treatment decreased (**Figure 1**, panels C and D; **Table 3**). Compared with non-smokers, the aHR of mortality for current smokers who smoked 30 or more cigarettes per day at baseline was 2.943 (95% CI = 1.035–8.368, *P* = 0.043), and 2.117 (95% CI = 1.157–3.871, *P* = 0.015) for those who smoked 30 or more cigarettes per day at baseline but quit during treatment.

**Table 3:** aHRs for baseline smoking status, death, and TB recurrence by smoking cessation during treatment*

	Death		Recurrence	
**Variables by category**	**aHR (95%CI)**	***P*-value**	**aHR (95%CI)**	
Smoking status				
*Non-smoker*	ref		ref	
*Ex-smoker*	1.384 (0.847–2.263)	0.194	1.843 (0.963–3.529)	0.065
Quit smoking during TB treatment				
*Smokes ≤15 per day*	0.643 (0.155–2.670)	0.543	1.368 (0.415–4.505)	0.606
*Smokes 16–29 per day*	1.571 (0.887–2.782)	0.121	2.581 (1.429–4.662)	<0.05
*Smokes ≥30 per day*	2.117 (1.157–3.871)	<0.05	5.159 (2.802–9.496)	<0.05
Continued smoking during TB treatment				
*Smokes 16–29 per day*	1.747 (0.803–3.801)	0.159	3.404 (1.456–7.958)	<0.05
*Smokes ≥30 per day*	2.943 (1.035–8.368)	<0.05	6.488 (2.163–19.461)	<0.05
Omnibus tests	χ^2^ = 123.682; *P* < 0.001		χ^2^ = 79.347; *P* < 0.001	

aHR – adjusted hazard ratio, CI – confidence interval, ref – reference

*We included age, type of TB, smear test, pleurisy and patient management as control variables.

Lastly, we divided the ex-smokers according to their smoking history. Compared with non-smokers, there was no significant difference in the risk of death or recurrent TB among ex-smokers who had smoked for less than 25 years (**Figure 1**, Panels E and F; **Table 4**). In contrast, for those with a smoking history of 25 years or more, the risk of recurrence was similar to that of current smokers who smoked 20 cigarettes daily. Compared with non-smokers, the aHR of mortality for current smokers who smoked 16 to 29 cigarettes per day at baseline was 2.635 (95% CI = 1.456–4.768, *P* = 0.001), and 2.137 (95% CI = 1.037–4.405; *P* = 0.040) for ex-smokers who had a history of smoking for 25 years or more.

**Table 4:** aHRs for baseline smoking status, death, and TB recurrence by smoking cessation and history*

	Death		Recurrence	
**Variables by category**	**aHR (95%CI)**	***P*-value**	**aHR (95%CI)**	***P*-value**
Smoking status				
Non-smoker	ref		ref	
Quit smoking during TB treatment				
*Smoke ≤15 per day*	0.643 (0.155–2.671)	0.544	1.383 (0.420–4.556)	0.594
*Smokes 16–29 per day*	1.602 (0.903–2.842)	0.107	2.635 (1.456–4.768)	<0.05
*Smokes ≥30 per day*	2.136 (1.181–3.962)	<0.05	5.267 (2.859–9.735)	<0.05
Continued smoking during TB treatment				
*Smokes 16–29 per day*	1.743 (0.799–3.804)	0.163	3.387 (1.451–7.909)	<0.05
*Smokes ≥30 per day*	3.036 (1.064–8.659)	<0.05	7.495 (2.509–22.386)	<0.05
Ex-smoker				
*Smoking history <25 y*	0.778 (0.229–2.649)	0.688	0.685 (0.091–5.144)	0.713
*Smoking history ≥25 y*	1.209 (0.687–2.128)	0.510	2.137 (1.037–4.405)	<0.05
Omnibus tests	χ^2^ = 107.724; *P* < 0.001		χ^2^ = 84.252; *P* < 0.001	

aHR – adjusted hazard ratio, CI – confidence interval, ref – reference

*We included age, type of TB, smear test, pleurisy and patient management as control variables.

Additionally, we identified a dose-response relationship between smoking frequency and the risk of death and TB recurrence, with a more significant effect on TB recurrence. Most of the slopes of the fitted lines are greater than 0 and have a *P*-value <0.05 (**Figure 1**, panels B, D, and F).

## DISCUSSION

To our knowledge, this is the first study to evaluate how smoking status and smoking cessation efforts affect both TB recurrence and mortality through long-term observation. As we hypothesised, compared with those who continued smoking, the risk of death and TB recurrence significantly decreased for patients who quit smoking during treatment due to the cessation intervention programme. Although this trend was stronger for heavy smokers, the positive effects were significant for most smokers who successfully quit smoking during TB treatment. Therefore, our findings align with those of previous studies [11,12].

The findings from this research provide further evidence that cigarette smoking is an independently confirmed risk factor for death and TB recurrence. This has implications for both policy and research in general. First, with the launch of the WHO’s ‘End TB Strategy’ targets for 2035 and the global effort to provide TB care and prevention, the global TB death rate gradually decreased until 2019. However, this trend has changed due to the negative impact of the coronavirus disease 2019 (COVID-19) pandemic, resulting in a need for urgent action to mitigate the increase in the absolute number of TB deaths. As cigarette smoking increases the risk of unfavourable treatment outcomes, delayed sputum smear/culture conversion, death, and recurrence, smoking cessation intervention should be integrated into routine TB services – an approach that requires discussion among policymakers.

Furthermore, we found a dose-response relationship between smoking frequency and the risk of TB mortality and recurrence during long-term follow-up, which also follows the findings from similar studies [15]. Other researchers also proposed the potential dose-response relationship exists between smoking amount and duration and the risk of recurrence [12]. Further studies reported that TB risk increased with the number of cigarettes smoked by the family members per day [16] or with both the daily dose of cigarettes and the duration of smoking [17].

Another notable finding was that ex-smokers with a history of smoking for more than 25 years had a high risk of death and TB recurrence. This can be explained by the fact that long-term smoking causes cumulative irreversible harm to health, especially to the respiratory system [18]. Therefore, reliable data on patient smoking status is crucial for guiding the risk assessment of death and TB recurrence. National TB programmes should consider this group in their active case-finding strategies.

This study has several limitations. First, we did not perform systematic medical examinations. We therefore do not know whether anyone had comorbidities, other risk factors or determinants, or how these factors were managed and further influenced death and TB recurrence. Second, instead of referring patients to a professional cessation clinic, cessation support was provided by trained TB staff at TB clinics. We therefore have no information on whether the patients received the same advice and support in terms of component, duration and frequency of interaction. Third, we did not revert to patients on findings from this research, a point that could improve the relevance and acceptability of the research findings and interventions. Additionally, smoking status was based on self-reports and not verified through biochemical tests, introducing the possibility of misclassification bias and undermining the accuracy of the results. However, a study using plasma cotinine as a reference standard has shown that self-reports are a reliable measure of smoking status [19]. Furthermore, even though we conducted this study in routine programme settings without any severe case or terminal illness, and although only 10 (4.0%) current smokers declined to participate at baseline, there may be a selection bias due to our study design. Lastly, we did not collect socioeconomic data, which could possibly be related to both death and TB recurrence.

Despite these limitations, this study has practical implications. First, smoking cessation interventions have neither been integrated into current routine TB care nor have they attracted attention from policymakers globally. Our findings suggest that brief advice and simple cessation support offered by trained staff in routine TB programme settings are effective in preventing death and TB recurrence beyond the completion of TB treatment. This supports further conduct of similar research in countries with comparable situations and provides evidence to support the WHO’s call for co-management of smoking and other risk factors as part of routine TB treatment [20]. Additionally, we suggest that TB service providers include smoking status and smoking history in their records and reporting systems. Therefore, a system with a standard data set is needed that enables health workers to input smoking information, as are governmental policies that would ensure that health institutions routinely collect such information. This approach will not only help promote standardised smoking cessation support but also create opportunities to identify at-risk populations during the monitoring of TB recurrence after the completion of anti-TB treatment.

## CONCLUSIONS

Using prospective cohort data, we confirmed the link between baseline smoking status and the increased risk of death and TB recurrence. Furthermore, quitting smoking during TB treatment significantly reduced the risk of death and TB recurrence during long-term follow-up. With the launch of the United Nations’ High-Level Meeting on TB Control Targets, it is important for countries with a high dual burden of TB and smoking to determine the most effective strategies for controlling these public health problems. Given the negative influence of the COVID-19 epidemic on TB mortality, cost-effective action is urgently needed to mitigate the current trend of increasing TB mortality in low- and middle-income countries. One effective approach is to incorporate effective smoking cessation intervention into routine TB services.
